# Parasites reduce food web robustness because they are sensitive to secondary extinction as illustrated by an invasive estuarine snail

**DOI:** 10.1098/rstb.2008.0220

**Published:** 2009-06-27

**Authors:** Kevin D. Lafferty, Armand M. Kuris

**Affiliations:** 1 U.S. Geological Survey, Western Ecological Research Center, c/o Marine Science Institute, University of CaliforniaSanta Barbara, CA 93106, USA; 2 Department of Ecology, Evolution and Marine Biology, University of CaliforniaSanta Barbara, CA 93106, USA

**Keywords:** food web, connectance, ecological analogue, subwebs, stability, trematode

## Abstract

A robust food web is one in which few secondary extinctions occur after removing species. We investigated how parasites affected the robustness of the Carpinteria Salt Marsh food web by conducting random species removals and a hypothetical, but plausible, species invasion. Parasites were much more likely than free-living species to suffer secondary extinctions following the removal of a free-living species from the food web. For this reason, the food web was less robust with the inclusion of parasites. Removal of the horn snail, *Cerithidea californica*, resulted in a disproportionate number of secondary parasite extinctions. The exotic Japanese mud snail, *Batillaria attramentaria*, is the ecological analogue of the native California horn snail and can completely replace it following invasion. Owing to the similarities between the two snail species, the invasion had no effect on predator–prey interactions. However, because the native snail is host for 17 host-specific parasites, and the invader is host to only one, comparison of a food web that includes parasites showed significant effects of invasion on the native community. The hypothetical invasion also significantly reduced the connectance of the web because the loss of 17 native trematode species eliminated many links.

## 1. Introduction

One way to measure the stability of a network, such as the Internet, is to simulate how the network responds to the addition or removal of nodes (Albert *et al*. 2000). For instance, in food webs, removing all of a consumer's resources will lead to its indirect extinction. Such secondary extinctions are more likely to result from the extirpation of highly connected resource species, especially prey species that have many predators (Sole & Montoya 2001; Allesina & Bodini 2004; Quince *et al*. 2005). Here, we ask how patterns of secondary extinction might change if we include parasites in food webs. We find that the high host specificity of parasites makes them very susceptible to extinctions of free-living species.

The Carpinteria Salt Marsh (CSM) estuary food web (Lafferty *et al*. 2006*b*) is a topological web that, similar to most other food webs, depicts links between predators and prey (87 free-living species). This food web is expandable to include 47 parasite species at CSM, making it currently the food web with the most complete inclusion of parasites. Parasites greatly increase connectance in the CSM food web (Lafferty *et al*. 2006*a*). We first compared how parasites and predators varied in their resource specialization and their subsequent sensitivity to secondary extinction. We also compared the robustness of the food web with and without parasites. Because the loss of one host species, the California horn snail, *Cerithidea californica*, led to a disproportionate number of secondary parasite species extinctions, we investigated a plausible scenario for the extirpation of the California horn snail from the CSM. The Japanese mud snail, *Batillaria attramentaria*, was inadvertently introduced before 1935 to several bays and estuaries along the Pacific coast of North America with shipments of the Pacific oyster, *Crassostrea gigas* (Carlton 1979; Byers 1999; Torchin *et al*. 2005; Miura *et al*. 2006). Discontinuous populations of the Japanese mud snail presently occur in estuaries from Boundary Bay, British Columbia, to Elkhorn Slough, California, overlapping with the northern range of the ecologically similar native horn snail. Where the invader and native overlap, the native has disappeared, or is declining (Carlton 1979; Byers 1999). Both snails graze on epipelic diatoms (competing exploitatively for food) and serve as the first intermediate hosts for trematodes (Martin 1972; Whitlatch & Obrebski 1980; Byers 2000; Miura *et al*. 2005; Torchin *et al*. 2005). Additionally, shared physiological requirements, direct development (Whitlatch 1972; Yamada-Behrens & Sankurathri 1977) and limited adult mobility (Sousa 1990) leave native snails few refugia in the presence of the invader (Byers 2000). Although replacement of the California horn snail by such a close ecological analogue would not be expected to have dramatic effects on the predator–prey links in an estuarine food web, it did result in the extirpation of numerous native trematode species from Elkhorn Slough, south of Santa Cruz, CA (Torchin *et al*. 2005). An undescribed heterophyid trematode morphospecies, *Cercaria batillariae*, reported from Japan successfully invaded all the West Coast populations of the Japanese mud snail, and is now well established. We compared topological properties of the CSM food web before and after a hypothetical invasion by the Japanese mud snail and a subsequent extinction of the native horn snail.

## 2. Material and methods

We used the CSM food web (Lafferty *et al*. 2006*b*) to examine the stability of the web with and without parasites. One indication of stability is to determine the pattern of dependencies in the network. Parasites with complex life cycles are likely to be more prone to extinction than a superficial analysis would indicate because each life stage has a potentially separate list of hosts. In other words, a parasite with a three-host life cycle would go extinct if any of the three hosts were absent, while investigation of the topological network would suggest that the parasite could persist as long as any of the three hosts were present. For this reason, we determined the ‘critical diet breadth’ of each species (species with broader diets being more buffered from secondary extinction). For free-living species, critical diet breadth was simply the number of prey species it consumed. For a parasite with a complex life cycle, each life stage has its own critical diet breadth. We determined the critical diet breadth of each parasite species as the life stage with the narrowest host range (the most specific stage in the parasite's life cycle). The extinction of the host(s) for this life stage, as opposed to other stages with a broader host range, would be most likely to lead to the secondary extinction of the parasite.

We also considered how the loss of each free-living species might lead to secondary extinctions of free-living and parasitic consumers. For instance, we removed the first free-living species, and counted the number of consumers that depended solely on it. We then replaced all species in the matrix, and removed the second free-living species. Here, and elsewhere, we did not remove detritus or carrion because we assumed these food sources would be invulnerable to extinction. We then created frequency histograms of the number of secondary extinctions (including cascading tertiary extinctions, etc.) per single primary extinction, separating parasites from free-living species.

We then measured robustness of the CSM food web to secondary extinctions. We evaluated robustness as the per cent of species that could be removed from the matrix before more than 50 per cent of the species became extinct via either direct removal or from secondary extinction (Dunne *et al*. 2002). We first removed species at random, then in order of their number of links. We then compared robustness for the web with only free-living species and the web with free-living and parasitic species.

To simulate a hypothetical invasion by the Japanese mud snail, we modified the CSM food web by replacing the California horn snail with the Japanese mud snail, eliminating the 17 trematode species in the California horn snail, and adding the introduced heterophyid trematode morphospecies. We note that the introduced populations of *C. batillariae* include three genetically recognizable species, one of which is rare (Miura *et al*. 2006). One of the common genetic species apparently invaded along with the snail, while the other was apparently able to invade America by dispersing with migratory birds that serve as the worm's final host (Miura *et al*. 2006). Unpublished genetic analyses of trematodes from *C. californica* also indicate the potential for several cryptic species. Because this level of taxonomy is not yet well resolved, we worked with the known, described species. However, to be certain that this did not affect the qualitative nature of our results, we ran a subsequent analysis under an assumption that would bias against the outcome that the invasion of the Japanese snail reduced trematode diversity. Here, we added to Japanese trematode diversity by including all three cryptic Japanese species but did not add cryptic native trematodes. After updating the corresponding changes in the links within the food web, we compared aspects (parasites per host and connectance) of these food webs and their subwebs before and after the invasion (and the extinction of the native snail).

To pinpoint the effects of invasion of the Japanese mud snail, we did a sensitivity analysis to observe how food-web properties would change according to different invasion scenarios in four independent variables: (i) competitive exclusion (or not) of the California horn snail, (ii) addition or non-addition of one generalist trematode, (iii) escape or lack of escape from native predators, and (iv) escape or lack of escape from native trematodes. This resulted in 16 unique outcomes each of which we simulated in a hypothetical web. The outcome that corresponded to an invasion of the Japanese mud snail to CSM was competitive exclusion of the California horn snail, addition of a generalist trematode, the lack of escape from native predators by *B. attramentaria* and escape from native trematodes (this outcome also corresponded to invasions of northern estuaries in the range of *C. californica*).

## 3. Results

**Figure 1:**
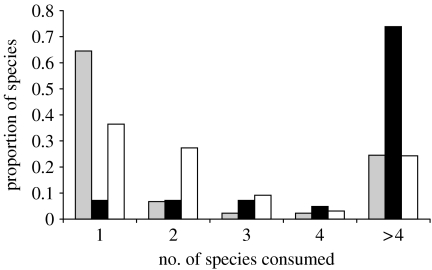
Frequency distribution of resource specialization for predators, parasites and scavengers expressed as the number of feeding links from the CSM food web. The horizontal axis categorizes species by how many feeding links they had (a measure of diet specialization). The vertical axis is the frequency of each feeding strategy that falls with each specialization bin. The figure illustrates how most parasites (grey bars) use only one host, while most predators (black bars) feed on several. Scavengers had a greater range of specialization (detritivores, white bars). Specialization should increase the risk of secondary extinction (figure 2).

Most parasites (64%) in the CSM web depended on a single host species at some point during their life cycle, while few predators depended on a single species (figure 1). Three trematode species were particularly sensitive to secondary extinctions because they depended on a single host species at two stages of their life cycle. A picornavirus was also dependent on the presence of two species because it is parasitic on a parasitic isopod, which has a single crab species for a host. The free-living species dependent on a single resource were either detritivores, carrion feeders or filter feeders of phytoplankton—all dietary items that aggregate many species and are very unlikely go extinct from the loss of a particular species in the food web.

**Figure 2:**
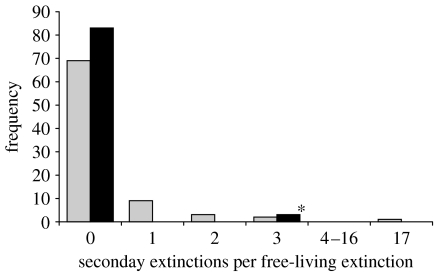
The distribution of secondary extinctions for predators (black bars) and parasites (grey bars) resulting from the primary extinction of each free-living species in the food web. Usually, free-living species were not the sole resource for a parasite or predator, so a few secondary extinctions resulted. The asterisk indicates three secondary extinctions of filter feeders following the extinction of phytoplankton. Because it is unlikely that the entire phytoplankton community would go extinct, these secondary extinctions seem unlikely. The distribution of secondary extinctions had a long right tail, indicating that the extinction of some hosts led to the extinction of one or more parasite species. The extinction of the snail *C. californica* led to the extinction of 17 trematode species.

Very few free-living species suffered secondary extinctions. By contrast, it was not unusual for parasites to suffer secondary extinctions. In 18 per cent of the cases, the extinction of a free-living species led to the secondary extinction of at least one parasite species (figure 2). Most secondary extinctions were of single parasite species, although sometimes several parasites simultaneously suffered secondary extinctions. In particular, extinction of the snail *C. californica* led to the secondary extinction of 17 trematode species.

Without parasites, the CSM food web was very robust to random species extinctions. Nearly half the time robustness was at its maximum value of 50 per cent. The median robustness was 49 per cent and the minimum robustness in 100 random trials was 44 per cent. The biggest decrease in robustness derived from the assumption that the phytoplankton community operated as a single species.

**Figure 3:**
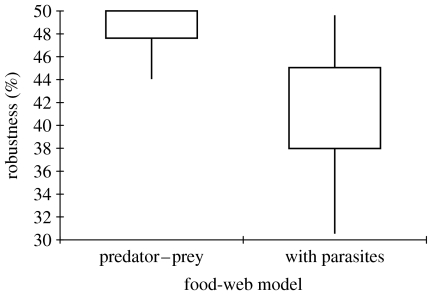
Robustness declines after adding parasites. The higher sensitivity of parasites to secondary extinction (figure 1) reduced the robustness of the food web. The variance in robustness was high after including parasites. This resulted from the skewed distribution in the distribution of secondary extinctions among free-living species (figure 2), particularly for the horn snail, *C. californica*. If the horn snail was included in the primary extinctions, 17 trematode species suffered secondary extinction.

Adding parasites reduced the robustness of the food web (figure 3). The median robustness was 43 per cent with a maximum of 49 per cent and a minimum of 30 per cent. Declines in robustness were the greatest if the horn snail was one of the species that was removed at random. Removing species in order of their linkage density (most linked species removed first) provided results that were only slightly different from removing species at random: median robustness was 48 per cent in the free-living web and 44 per cent in the web with parasites.

**Figure 4:**
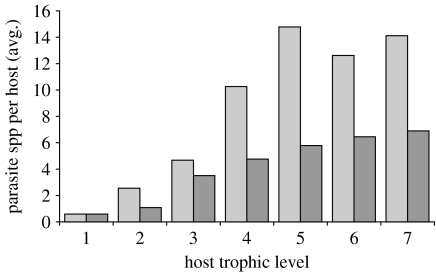
Change in the average number of parasite species per host species in CSM associated with the hypothetical invasion of the Japanese mud snail. Pre-invasion data and trophic-level calculation from Lafferty *et al*. (2006*a*; light grey bars, pre-invasion; dark grey bars, post-invasion).

Because the California horn snail and the Japanese mud snail are ecological analogues, our conceptual introduction followed by the extinction was assumed to have no effect on the predator–prey subweb at CSM. Most food-web studies have quantified only the predator–prey subweb, and doing so gave the impression this invasion would be without consequence for CSM. The loss of 17 native trematode species and the gain of only one exotic trematode would result in an obvious net loss of biodiversity by 16 species. Because trematodes have complex life cycles, their absence would have resonated throughout the ecosystem in that many free-living species would have experienced less parasitism (Torchin *et al*. 2005) and to the connectance of those food webs, as well as provided substrate. Only half (274/615) of the parasite–host subweb links would have remained, resulting in a dramatic decrease in the average number of parasite species per host (figure 4). Half (480/1021) of the predator–parasite subweb links and a very few (3/172) of the parasite–parasite subweb links would be retained. In total, links in the food web would have declined by half (1262/2313).

**Figure 5:**
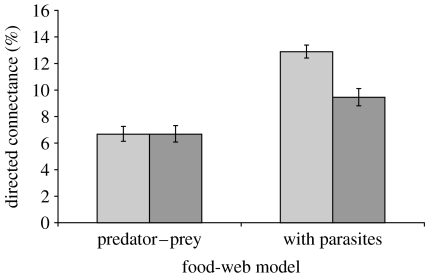
Change in connectance in CSM associated with a hypothetical invasion of the Japanese mud snail. After invasion, directed connectance was unchanged if parasites were not considered. Considering parasites in the food web led to a large decline in connectance following invasion, indicating that traditional food-web studies would fail to see an effect of the Japanese mud snail on food-web properties (light grey bars, pre-invasion; dark grey bars, post-invasion).

Directed connectance would not have changed in the predator–prey subweb (6.7%) but would have declined in the complete food web if we considered the effect on parasites (from 13 to 9%; figure 5). The sensitivity analysis indicated that this would have been due primarily to the high specificity of the native trematodes for the extirpated first intermediate host. The invasion of the Japanese trematode would have done little to make up for the loss of several native species in terms of connectance. Furthermore, including cryptic Japanese trematode species in the invaded web did not change the qualitative results.

## 4. Discussion

It is important to emphasize that the reduction in robustness caused by the inclusion of parasites did not result from parasite-induced extinctions of hosts (an outcome not possible in our topological approach). Instead, the decrease in robustness was due to the higher sensitivity of parasites to secondary extinction. This finding was only possible when considering that each life stage in a trematode life cycle has a potentially different set of hosts. Had we simply lumped all life stages, the trematodes would have appeared to have had a wide host range and to have been relatively invulnerable to secondary extinction.

Empirical studies in this system reveal more subtle dependencies of parasites on the host community. For instance, a decrease in the diversity and abundance of birds at a particular site directly decreases the diversity and abundance of trematodes using *C. californica* (Hechinger & Lafferty 2005). Furthermore, the trematode assemblage at a particular location depends on the assemblages of fishes and invertebrates that serve as second intermediate hosts to these trematodes. As a result, habitat degradation can reduce the diversity and abundance of trematodes in snails; conversely, habitat restoration can help recover these parasite communities (Huspeni & Lafferty 2004).

Invasion of CSM by the Japanese mud snail is a hypothetical but realistic scenario. In addition, our results probably pertain to actual extinctions because the food web of CSM overlaps substantially with estuaries where the Japanese mud snail has replaced (Elkhorn Slough) or is in the process of replacing the California horn snail (Bolinas Lagoon, Tomales Bay). Other estuaries (Padilla Bay, Boundary Bay, Willapa Bay) invaded by the Japanese mud snail are north of the range of the California horn snail (Byers 1999). In those bays, the invader did not replace an ecological analogue and presumably its invasion resulted in a net gain in the parasite fauna (Torchin *et al*. 2005) and in the connectance of those food webs, as well as providing substrate (shells) for other invaders (Wonham *et al*. 2005). For this reason, the effects of this invasion on food-web structure are highly context dependent.

Our results expand on the finding that the invasion of the Japanese mud snail reduces parasitism on various hosts in invaded estuaries (Torchin *et al*. 2005). Whether the topological changes we see in our hypothetical invasion translate into equally dramatic changes in energy flow through the system depends on the interaction strengths of the links lost and gained.

One possible consequence of lost parasite links relates to the ability of parasites to alter predator–prey interactions. Various trematodes from the California horn snail commonly use six species of fishes, seven crustaceans, three annelids and six molluscs as second intermediate hosts at CSM (Huspeni & Lafferty 2004). A dramatic reduction in trematode diversity could favour some free-living species over others, with invertebrates being the main beneficiaries. One of the most common trematode species, *Euhaplorchis californiensis*, affects the behaviour of the California killifish, *Fundulus parvipinnis*, predisposing the fish to predation by birds that serve as final hosts (Lafferty & Morris 1996). It seems probable that the killifish in CSM would benefit from the extinction of the native *E. californiensis*. The effects of other trematodes are unknown, although we suspect that some of them could also facilitate predation to final hosts. Trematode metacercariae are known to affect the behaviour of crustacean (Helluy 1983) and bivalve (Thomas & Poulin 1998) hosts in ways that increase the risk of predation. The loss of such parasites could reduce the intensity of some predator–prey interactions, leading to substantial changes in the interactive properties of the predator–prey subweb. Loss of species that are pathogenic might benefit certain bird species. However, since some trematodes facilitate predation, and may not be very pathogenic, the loss of parasites that facilitate predation by birds could be a significant cost for final host populations (Lafferty 1992).

Whether the addition of the Japanese trematodes could make up for these losses is not known at this time. Metacercariae of the Japanese trematodes use fishes but not the 16 invertebrates as second intermediate hosts. It is unclear how the exchange of several native heterophyids for a Japanese heterophyid would alter the impact of parasitism on most fishes. This would depend, in part, on if and how the Japanese trematode affects fish behaviour and fitness.

The decrease in connectance following invasion required the assumption that invasion by the Japanese snail led to a complete replacement of the native snail. This is not the rule in invasion biology, and we caution that our topological results will not apply to all invasion scenarios. If *B. attramentaria* did not lead to complete exclusion of the native *C. californica*, the increase in trematode diversity would have led to an increase in connectance. Our hypothetical invasion of *B. attramentaria* was one way to probe the robustness of parasites in food webs; however, other situations can also result in the absence of *C. californica* from salt marsh food webs. The subtropical genus *Cerithidea* is absent from northern latitudes. It is also intolerant of the non-tidal conditions common to small estuaries or to the low salinities present in river-mouth estuaries (K.D. Lafferty 2004, personal observations). This peculiar distribution of *C. californica* provides the potential to examine the robustness of many replicated food webs with and without a major parasitological component.

We suspect that parasites, on average, will be more sensitive to secondary extinction compared with free-living species. This is due to their relative propensity to specialize on one or a few host species at each life stage. Adding extinction-prone parasites to food webs, therefore, reduces the robustness of the entire web. While few will mourn the loss of parasites, this feature may make parasites excellent indicators of food-web integrity. For this system, trematode communities are excellent indicators of the abundance and diversity of final hosts (birds: Hechinger & Lafferty 2005; mammals: Lafferty & Dunham 2005) and also indicate the aspects of the second intermediate host communities (fishes and invertebrates: Hechinger *et al*. 2007); even being able to track degradation and recovery of estuary habitat over time (Huspeni & Lafferty 2004).
